# Androgen receptor pathway inhibitors vs. docetaxel chemotherapy for metastatic hormone-sensitive and first-line castration resistant prostate cancer

**DOI:** 10.1007/s00345-024-05388-1

**Published:** 2024-12-28

**Authors:** Mike Wenzel, Benedikt Hoeh, Clara Humke, Cristina Cano Garcia, Carolin Siech, Thomas Steuber, Markus Graefen, Miriam Traumann, Luis Kluth, Felix K. H. Chun, Philipp Mandel

**Affiliations:** 1 Department of Urology, University Hospital Frankfurt, Goethe University Frankfurt am Main, Frankfurt, Germany; 2 Martini-Klinik Prostate Cancer Center, University Hospital Hamburg-Eppendorf, Hamburg, Germany

**Keywords:** mHSPC, mCRPC, high volume, Apalutamide, ARPI, Enzalutamide

## Abstract

**Purpose:**

No currently available phase III trial compared docetaxel vs. androgen receptor pathway inhibitors (ARPI) regarding cancer-control outcomes in metastatic hormone-sensitive prostate cancer (mHSPC). Moreover, few is known about the effect of sequential therapies in mHSPC and subsequent metastatic castration resistant prostate cancer (mCRPC).

**Methods:**

We relied on the FRAMCAP database and compared docetaxel vs. ARPI in mHSPC patients regarding time to mCRPC (ttCRPC) and overall survival (OS). Sensitivity analyses addressed high volume mHSPC patients. Finally, sequential therapies were compared regarding progression-free survival (PFS) and OS in first-line mCRPC.

**Results:**

Of 419 included mHSPC patients, 25% received docetaxel vs. 75% ARPI. ARPI patients were significantly older (71 vs. 66 years), and harbored lower baseline PSA (38 vs. 183 ng/ml, both *p* ≤ 0.002). Median ttCRPC was significantly longer for ARPI than for docetaxel-treated patients (30 vs. 17 months, hazard ratio [HR]: 0.49, *p* < 0.001). In OS analyses, ARPI patients also exhibited significantly longer OS, relative to docetaxel patients (96 vs. 50 months, HR: 0.67, *p* = 0.03). After multivariable adjustment in Cox regression models, no difference between both treatments remained in both analyses (all *p* > 0.05). In sensitivity analyses of high volume mHSPC patients only, also no ttCRPC or OS differences were observed for ARPI vs. docetaxel (all *p* > 0.05). Regarding sequential therapies, no PFS and OS differences were observed for all and specifically high volume mHSPC patients, when ARPI-ARPI vs. ARPI-docetaxel vs. docetaxel-ARPI treatments were compared (all *p* > 0.05).

**Conclusion:**

In real-world setting, ARPI treatment performs comparable to docetaxel chemotherapy in mHSPC. Therefore, docetaxel should only be used in triplet therapy. Moreover, no differences for sequential therapies of ARPI/docetaxel combinations in first-line mCRPC were observed.

**Supplementary Information:**

The online version contains supplementary material available at 10.1007/s00345-024-05388-1.

## Introduction

Androgen deprivation monotherapy (ADT) has been the standard of care in the treatment for metastatic hormone-sensitive prostate cancer (mHSPC) and metastatic castration-resistant prostate cancer (mCRPC) for decades [1]. With the approval of additional combination therapies such as docetaxel chemotherapy or androgen receptor pathway inhibitors (ARPI) providing substantially longer overall survival (OS) in mHSPC, the gold standard currently remains a combination therapy of ADT plus docetaxel or ARPI for mHSPC and mCRPC or both combined for mHSPC as triplet therapy [2–12].

However, some combination therapies only improved OS in specific mHSPC patient cohorts. Specifically, in the CHAARTED trial, docetaxel improved OS only in high volume mHSPC patients [2, 13]. Similarly, in the LATITUDE trial only patients with high-risk mHSPC were included [3]. Conversely, enzalutamide or apalutamide improved OS irrespective of metastatic burden [4–7]. In clinical practice, clinicians therefore currently sought for best first-line mHSPC treatment selection. Some previous network metanalyses suggested better cancer-control outcomes of ARPI treatment over docetaxel chemotherapy, especially in high-volume mHSPC patients [14, 15]. Moreover, a previous study statistically providing a weighted median out of all available phase III trials comparing docetaxel vs. abiraterone in high volume mHSPC also observed a five months OS advantage for abiraterone [16]. Nonetheless, currently no randomized phase III trial compared docetaxel chemotherapy vs. ARPI treatment for mHSPC patients. However, in the STAMPEDE trial, a head-to-head comparison between docetaxel and abiraterone did not yield significant differences in survival in advanced or metastatic prostate cancer [17]. Moreover, it currently remains unclear, how first-line mHSPC treatment and choice of subsequent first-line mCRPC treatment sequence affects OS outcomes.

We addressed these voids and relied on the FRAMCAP database (Frankfurt Metastatic Cancer Database of the Prostate) to provide head-to-head comparisons of mHSPC patients receiving docetaxel chemotherapy vs. ARPI treatment. We hypothesized that in real-world setting important differences in baseline characteristics and cancer-control outcomes exist between both treatments for mHSPC. Moreover, we also hypothesized that sequential treatment choice for primary mHSPC and subsequent mCRPC treatment substantially influence cancer-control outcomes.

## Materials and methods

### Study population

After approval of the local ethics committee (reference number: SUG-5-2024) and in accordance with the principles of the Declaration of Helsinki, we conducted a retrospective identification of all metastatic prostate cancer patients from the prospective sampling FRAMCAP (Frankfurt Metastatic Cancer database of the Prostate) database. All patients were treated at the Department of Urology, University Hospital Frankfurt, Germany (*n* = 1127). For analysis, only patients with ARPI or docetaxel treatment for mHSPC were included. These criteria yielded 419 eligible mHSPC patients.

### Statistical analysis

Descriptive statistics included the frequencies and proportions of categorical variables used in the analysis. Median values and interquartile ranges (IQR) were reported for all continuous variables. The Chi-square test was employed to evaluate the statistical significance of differences in proportions, while the t-test and Kruskal-Wallis test were used to analyze differences in distributions.

In the first step of analyses, time to mCRPC and OS outcomes were compared between mHSPC patients treated with docetaxel vs. ARPI. Subsequently, these cancer-control analyses were repeated in sensitivity analyses for high volume mHSPC patients only.

In the second step of analyses, progression-free survival (PFS) and OS outcomes were performed in mCRPC patients stratified according to initially received mHSPC and subsequent first-line mCRPC treatment. Here, three groups were compared, namely ARPI-ARPI vs. ARPI-docetaxel vs. docetaxel-ARPI treatment. Additionally, also sensitivity analyses were performed for mHSPC high volume patients.

For all cancer-control outcome comparisons, univariable, as well as multivariable Cox regression models were applied. Adjustment in multivariable Cox regression models were performed for age at mHSPC, PSA at mHSPC, Eastern Cooperative Oncology Group (ECOG) status, De Novo mHSPC, high volume mHSPC and year of diagnosis for PFS analyses and additionally for the number of received systemic treatment for OS analyses. All tests were two sided with a level of significance set at *p* < 0.05. R software environment for statistical computing and graphics (version 3.4.3) was used for all analyses.

## Results

Overall, 419 mHSPC patients qualified for final analyses, of which 25% (*n* = 105) received docetaxel vs. 75% (*n* = 314) ARPI treatment (Suppl. Table 1). Median age at mHSPC was 70 years (IQR: 63–75) with a median PSA of 46 ng/ml (IQR: 13–274). Overall, 4.8% of included patients were classified as ECOG status ≥ 2. Proportions of De Novo, high volume and visceral metastasis at mHSPC were 76%, 64% and 7.8%, respectively. Median follow up was 27 months (IQR: 11–46).

### mHSPC: Docetaxel vs. ARPI

In comparison between mHSPC patients treated with either docetaxel vs. ARPI (Suppl. Table 1), patients with ARPI were significantly older at mHSPC diagnosis (71 vs. 66 years), and harbored lower baseline PSA (38 vs. 183 ng/ml, both *p* ≤ 0.002), relative to docetaxel mHSPC patients. Regarding frailty status, no significant differences in ECOG status distribution, cardiovascular diseases or secondary malignancies were observed (all *p* ≥ 0.2). MHSPC patients receiving docetaxel harbored significantly higher rates of De Novo (89% vs. 71%), high volume mHSPC (80% vs. 49%) and visceral metastases (15% vs. 5.6%), than ARPI counterparts (all *p* ≤ 0.01). Regarding treatment responses, lower PSA nadir (0.10 vs. 0.65 ng/ml) and higher ≥ 99% PSA response (70% vs. 54%) were observed for ARPI vs. docetaxel (both *p* ≤ 0.027).

Regarding cancer-control outcomes, significant differences for all mHSPC patients were observed. Specifically, median time to mCRPC was significantly longer for ARPI than for docetaxel-treated patients (30 vs. 17 months, hazard ratio [HR]: 0.49, *p* < 0.001, Fig. 2A). In multivariable Cox regression models adjusting for baseline and tumor characteristics, no significant difference remained (HR: 0.96, *p* = 0.9, Suppl. Table 2A).

**Fig. 1:**
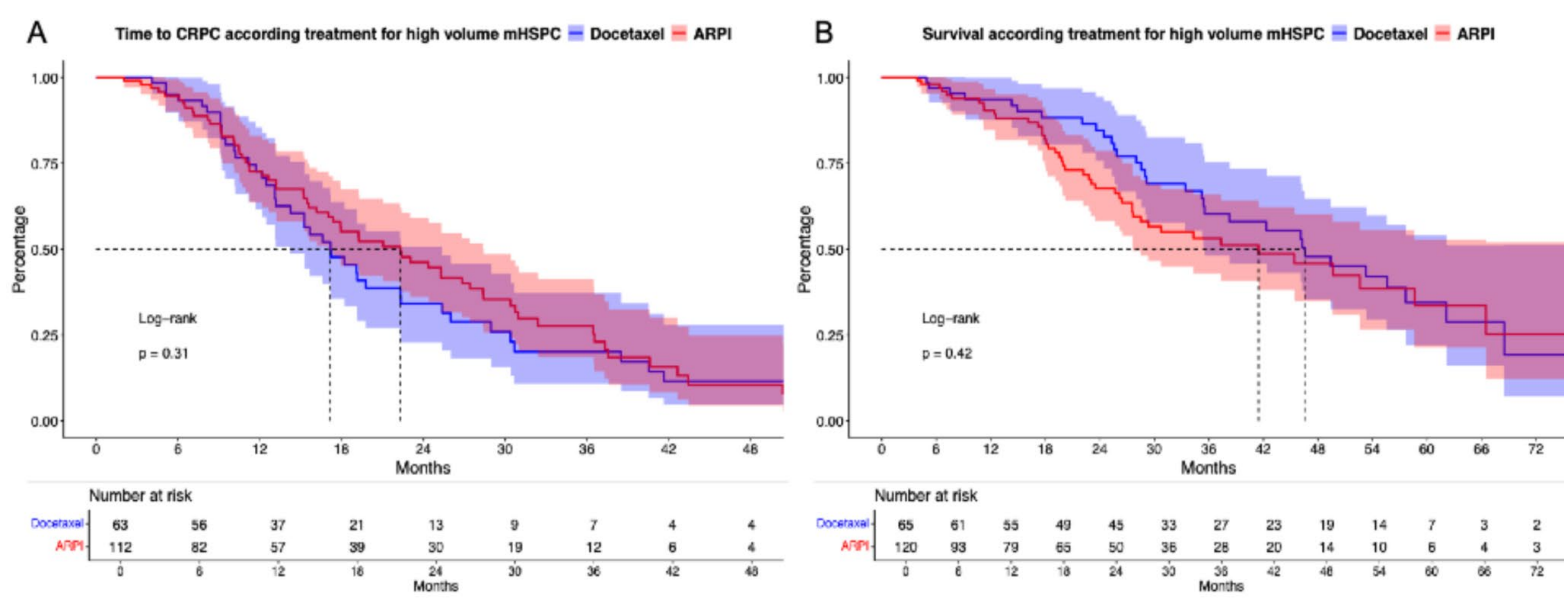
Kaplan Meier curves depicting time to metastatic castration resistant prostate cancer (CRPC, **A**) and overall survival (**B**) according treatment for high volume metastatic hormone-sensitive prostate cancer (mHSPC) stratified according to docetaxel vs. androgen receptor pathway inhibitor (ARPI)

In OS analyses, ARPI patients also exhibited significantly longer OS, relative to docetaxel patients (96 vs. 50 months, HR: 0.67, *p* = 0.03, Fig. 1B). After multivariable adjustment in Cox regression models, also no difference between both treatments remained (HR: 1.03, *p* = 0.9, Suppl. Table 2B).

**Fig. 2:**
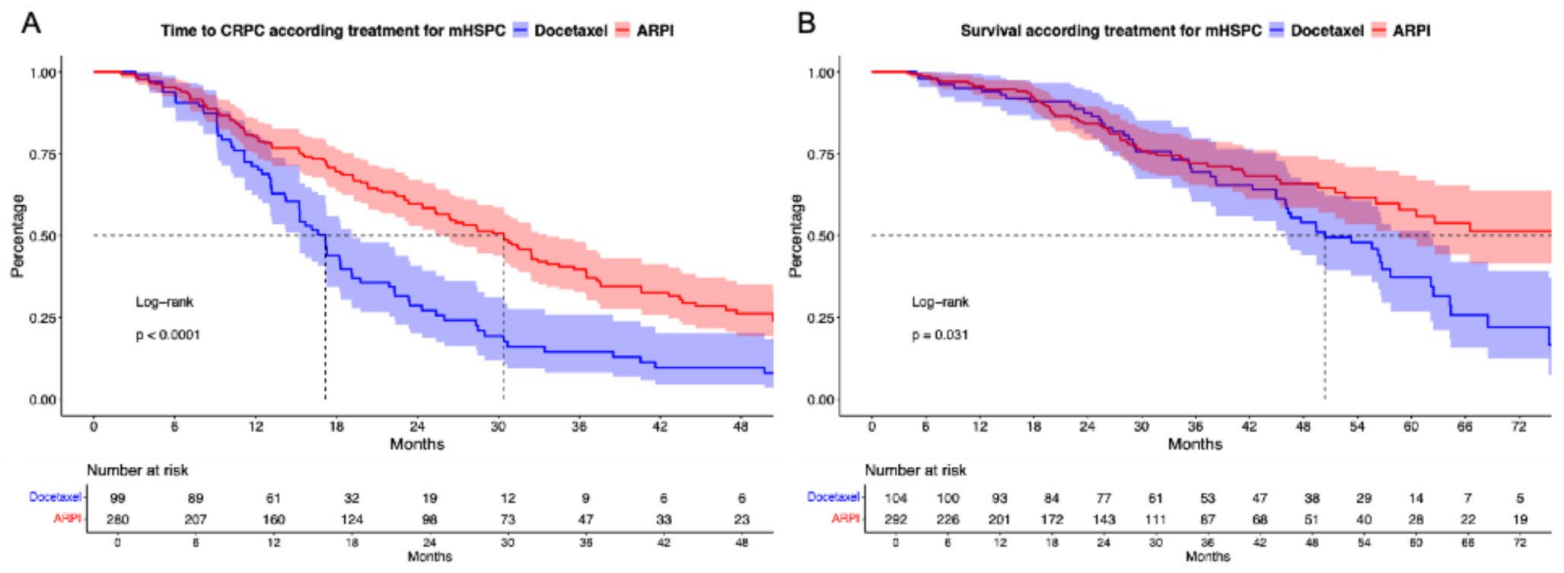
Kaplan Meier curves depicting time to metastatic castration resistant prostate cancer (CRPC, **A**) and overall survival (**B**) according treatment for metastatic hormone-sensitive prostate cancer (mHSPC) stratified according to docetaxel vs. androgen receptor pathway inhibitor (ARPI)

### High volume mHSPC: Docetaxel vs. ARPI

In sensitivity analyses of 192 high volume mHSPC patients, 34% (*n* = 65) received docetaxel vs. 66% (*n* = 127) ARPI treatment. Patients with ARPI were significantly older in median at mHSPC (72 vs. 67 years) and harbored lower baseline PSA (133 vs. 360 ng/ml, both *p* < 0.05). No difference in ECOG status distribution, cardiovascular diseases or secondary malignancy rates were observed (all *p* ≥ 0.12). Moreover, no difference in PSA nadir at mHSPC (0.54 vs. 0.88 ng/ml) or PSA ≥ 99% responses (64% vs. 60%) were observed for ARPI vs. docetaxel (both *p* ≥ 0.3). Additionally, no differences in rates of De Novo mHSPC (85% vs. 91%) or visceral metastasis (11 vs. 20%) were seen (both ≥ 0.1).

In time to mCRPC analyses (Fig. 2A), no significant difference between ARPI vs. docetaxel high volume mHSPC patients were observed (median time: 22 vs. 17 months, HR: 0.82, *p* = 0.3). Similarly in OS analyses (Fig. 2B), also no difference between ARPI and docetaxel were observed (median time: 42 vs. 46 months, HR: 1.21, *p* = 0.4). In both multivariable Cox regression models, no significant differences between both treatments could be computed (Suppl. Table 2A-B).

### Sequential therapies for mHSPC and first-line mCRPC

Sequential treatment information for 143 mHSPC with subsequent progress to mCRPC were available. Of these, 35% (*n* = 50) received ARPI-ARPI vs. 27% (*n* = 38) ARPI-docetaxel vs. 38% (*n* = 55) docetaxel-ARPI combination.

In PFS analysis from time point of progressing to mCRPC (first-line therapy for mCRPC), no significant differences for all three sequential therapies were observed with median time of 10.3 vs. 7.1 vs. 9.2 months for ARPI-ARPI vs. ARPI-docetaxel vs. docetaxel-ARPI combination (Suppl. Figure 1 A, *p* = 0.3). However, PFS2 was better for ARPI-ARPI over ARPI-docetaxel and docetaxel-ARPI after multivariable adjustment.

In OS analyses (Suppl. Figure 1B), no differences were observed with median OS of 67 vs. not reached vs. 57 months for ARPI-ARPI vs. ARPI-docetaxel vs. docetaxel-ARPI combination (*p* = 0.5). In landmark-analyses from time point of progressing to mCRPC (Suppl. Figure 1 C), also no OS differences were observed (*p* = 0.7). In all multivariable Cox regression models, no significant difference between all three compared sequential therapies were observed (Suppl. Table 2 C-D),

In subsequent sensitivity analyses of high volume mHSPC patients (Suppl. Figure 2 A-C), 67 patients qualified for analyses, of which 30% (*n* = 20) vs. 25% (*n* = 17) vs. 45% (*n* = 30) received ARPI-ARPI vs. ARPI-docetaxel vs. docetaxel-ARPI combination. Similar to all mHSPC patients, in high volume mHSPC no differences in PFS for first-line mCRPC treatment and OS analyses were observed for all three compared sequential therapies (all *p* ≥ 0.3). Finally in multivariable Cox regression models, no significant differences between all three sequential therapies were computed (Suppl. Table 2 C-D).

## Discussion

We hypothesized that in clinical real-world setting important differences in baseline characteristics and cancer-control outcomes exist between ARPI and docetaxel treated patients in mHSPC. We also hypothesized that sequential treatment choice for mHSPC and subsequent mCRPC treatment (first-line mCRPC) substantially influence cancer-control outcomes such as PFS and OS. We tested these hypotheses within the FRAMCAP database and made several important observations.

First, we observed significant differences in baseline and tumor characteristics between mHSPC patients treated with docetaxel vs. ARPI. More specifically, the majority of patients received ARPI (75%) for mHSPC treatment. Moreover, patients receiving ARPI were significantly older and harbored lower baseline PSA value at metastatic diagnosis. Conversely, docetaxel-treated mHSPC patients harbored higher rates of De Novo, high volume and visceral metastasis at mHSPC disease. However, no differences in comorbidities or ECOG status were observed between both groups. On the one hand, it is not surprising that patients with more favorable tumor characteristics (such as lower rates of high volume mHSPC) received ARPI treatment, since the proved OS advantage for docetaxel chemotherapy in mHSPC was especially observed in the CHAARTED trial for high volume mHSPC patients [2]. These unfavorable tumor characteristics such as high volume metastatic burden may also explain differences in baseline PSA values. Conversely, on the other hand, one may have expected that patients receiving docetaxel chemotherapy may harbor lower rates of comorbidities or frail performance status, relative to ARPI-treated mHSPC patients. These observations suggest that treatment selection was mainly based on favorable/unfavorable tumor characteristics and evidence from the CHAARTED trial instead of other clinical parameters. However, when sensitivity analyses of high volume mHSPC patients were performed, the observed differences in unfavorable tumor characteristics partly vanished (equalized balanced De Novo mHSPC and visceral metastasis proportions) and the proportion of docetaxel-treated mHSPC patients was higher than for all mHSPC patients (34% vs. 25%).

Second, we also made important observations regarding cancer-control outcomes in docetaxel vs. ARPI-treated mHSPC patients. Specifically, we found that time to mCRPC and OS was significantly longer in ARPI mHSPC patients, relative to docetaxel mHSPC patients. These observations are not surprising since docetaxel mHSPC patients harbored significant worse baseline tumor characteristics in comparison to ARPI treated patients, as discussed above. In order to maximally adjust and balance for these differences in baseline patient and tumor characteristics, we applied multivariable Cox regression models. In these multivariable models, the differences between both compared ARPI vs. docetaxel treatment disappeared. These observations indicate that the observed significant univariable time to mCRPC and OS differences are probably mainly based on patient selection. The observations also emphasize the almost equal potential of ARPI and docetaxel treatment in first-line mHSPC setting. These findings are in line with previous published cohorts. For example, in a propensity-score matched cohort of docetaxel and abiraterone-treated mHSPC patients, also almost similar three-year OS rates were observed for both treatments (76.2% vs. 75.1%) [18]. Additionally, virtually similar findings to our study were made in other previous reported studies, in which unadjusted time to mCRPC rates were better for abiraterone vs. docetaxel, but no OS differences were observed, like in the prospective STAMPEDE trial [17, 19–23]. However, most studies only focused solely on abiraterone and no previous head-to-heard comparison for enzalutamide or apalutamide vs. docetaxel are available. When using network meta-analyses combining and comparing data from all available phase III trials, also no difference in docetaxel vs. apalutamide or docetaxel vs. enzalutamide or docetaxel treated patients were mostly observed [15, 18, 24, 25].

Comparing data in sensitivity analyses of high volume mHSPC patients only, also no differences in time to mCRPC or OS analyses were observed in univariable, as well as multivariable Cox regression models were observed. These observations indicate the potential of ARPI treatment also in high volume mHSPC as equal to docetaxel chemotherapy. One clinical conclusion drawn from these findings may be that there is currently no clinical indication for docetaxel chemotherapy in patients with mHSPC, since equal effects can be achieved with ARPI plus ADT. When docetaxel chemotherapy is considered, it should be combined (with abiraterone or) darolutamide and ADT as a triplet therapy, for which OS advantages relative to ADT and docetaxel doublet therapy have been observed especially in high volume mHSPC patients [8, 9, 14, 15].

Finally, we compared sequential therapies for mHSPC patients with subsequent treatment for first-line mCRPC. Here, we observed that the most frequently administered combination was docetaxel-ARPI. However, no difference in PFS and OS analyses were observed for all patients and in sensitivity of high volume mHSPC patients only, regardless of the used sequential therapy (ARPI-ARPI vs. ARPI-docetaxel vs. docetaxel-ARPI). These observations are in line with previous reports. For example, in a report of 107 docetaxel and 233 abiraterone mHSPC patients by Ozaki et al., also PFS2 was comparable irrespective of primary mHSPC treatment [23]. In an American Health Care Record database, also no differences in PFS2 were observed for enzalutamide vs. docetaxel in first-line mCRPC after abiraterone in mHSPC setting, as well as for abiraterone vs. docetaxel in mCRPC after enzalutamide in mHSPC setting [26]. Conversely, another multicenter report by Tsaur et al. of 17 vs. 48 patients investigated that treatment switch to ARPI after initial docetaxel chemotherapy for mHSPC was associated with better OS [27]. However, in our study those PFS2 findings could not be replicated as PFS2 was significant longer in the ARPI-ARPI sequence. Nevertheless, these findings did not translate in any OS advantage, which may be explained by the effect of subsequent systemic treatments [28]. A possible explanation might be that PFS2 may be biased as treatment after progression is more common in patients with ARPI therapy compared to cytotoxic chemotherapy. Further prospective studies need to validate these inconclusive finding in order to maximally eliminate the potential bias of the retrospective design and potential selection bias.

Other limitations of the current study should also be acknowledged in in its interpretation. Some missing or not sampled variables may have influenced treatment outcomes, even multivariable adjustment was performed to maximally balance all compared groups. Moreover, patients treated with docetaxel upfront may represent more historical mHSPC patients, while ARPI patients may be more contemporary. In order to equalize for this effect, we adjusted all Cox models for the year of diagnosis. Finally, some subgroups are rather small for a reliable analysis.

Taken together, the current study shows equal cancer-control effects of docetaxel vs. ARPI treatment for all mHSPC and specific high volume mHSPC patients. These observations indicate that docetaxel should be only used in combination with ARPI and ADT as triplet therapy. Moreover, the current study also showed that all available sequential therapies in mHSPC and subsequent mCRPC (first-line mCRPC) provide almost similar cancer-control outcomes. However, these findings should be ideally validated within prospective trials, also accounting for subsequent systemic treatments.

## Electronic Supplementary Material

Below is the link to the electronic supplementary material.

Supplementary Material 1: Fig. 1. Kaplan Meier curves depicting progression-free survival (PFS, A) and overall survival (OS) in metastatic hormone-sensitive prostate cancer (mHSPC, B) and metastatic castration resistant prostate cancer patients (mCRPC, C) stratified according to sequential treatments with docetaxel (Doce) and androgen receptor pathway inhibitors (ARPI).

Supplementary Material 2: Fig. 2. Kaplan Meier curves depicting progression-free survival (PFS, A) and overall survival (OS) in metastatic hormone-sensitive prostate cancer (mHSPC, B) and OS in metastatic castration resistant prostate cancer patients (mCRPC, C) with initially high volume (HV) metastatic hormone-sensitive prostate cancer stratified according to sequential treatments with docetaxel (Doce) and androgen receptor pathway inhibitors (ARPI).

Supplementary Material 3: Table 1. Characteristics of 419 metastatic hormone-sensitive prostate cancer (mHSPC) patients stratified according to treatment with docetaxel vs. androgen receptor pathway inhibitor (ARPI). Abbreviations: PSA: Prostate-specific antigen, mCRPC: metastatic castration-resistant prostate cancer, ECOG: Eastern Cooperative Oncology group, CVD: Cardiovascular disease, Ca: Cancer, RP: Radical prostatectomy, RT: Radiation therapy, Lu-PSMA: Lutetium-Radioligand therapy, NA: Unknown.

Supplementary Material 4: Table 2. Univariable und multivariable Cox regression models predicting time to metastatic castration resistant prostate cancer (mCRPC; A) and overall survival (OS; B) in metastatic hormone-sensitive prostate cancer (mHSPC) patients and progression-free survival (PFS, C) and OS (D) regarding sequential therapies in mCRPC: Abbreviation: HR: Hazard Ratio, CI: Confidence interval, androgen receptor pathway inhibitor (ARPI), ECOG: Eastern Cooperative Oncology Group.

## Data Availability

Data are available for bona fide researchers who request it from the authors.
