# Supplementary material for: Androgen receptor pathway inhibitors vs. docetaxel chemotherapy for metastatic hormone-sensitive and first-line castration resistant prostate cancer
Source: World J Urol. 2024 Dec 28;43(1):51. doi: 10.1007/s00345-024-05388-1 (PMC11682002; doi:10.1007/s00345-024-05388-1)
Supplement: Supplementary file 3 — Supplementary Material 3: Table 1. Characteristics of 419 metastatic hormone-sensitive prostate cancer (mHSPC) patients stratified according to treatment with docetaxel vs. androgen receptor pathway inhibitor (ARPI). Abbreviations: PSA: Prostate-specific antigen, mCRPC: metastatic castration-resistant prostate cancer, ECOG: Eastern Cooperative Oncology group, CVD: Cardiovascular disease, Ca: Cancer, RP: Radical prostatectomy, RT: Radiation therapy, Lu-PSMA: Lutetium-Radioligand therapy, NA: Unknown. [file 345_2024_5388_MOESM3_ESM.docx]

| **Characteristic** | ****N**** | ****Overall****,  N = 419*^1^* | ****Docetaxel****,  N = 105 (25%)*^1^* | ****ARPI****,  N = 314 (75%)*^1^* | ****p-value*****^2^* |
| --- | --- | --- | --- | --- | --- |
| Age at mHSPC | 400 | 70 (63, 75) | 66 (61, 71) | 71 (64, 77) | <0.001 |
| PSA at mHSPC, ng/ml | 317 | 46 (13, 274) | 183 (20, 631) | 38 (11, 167) | <0.001 |
| PSA nadir mHSPC, ng/ml | 225 | 0.17 (0.02, 1.2) | 0.65 (0.13, 3.32) | 0.10 (0.01, 0.73) | <0.001 |
| PSA response ≥99% | 203 | 133 (66%) | 30 (54%) | 103 (70%) | 0.027 |
| PSA at mCRPC, ng/ml | 128 | 16 (4, 75) | 12 (3, 53) | 18 (6, 81) | 0.4 |
| ECOG status | 315 |  |  |  | 0.2 |
| 0 |  | 207 (66%) | 47 (58%) | 160 (68%) |  |
| 1 |  | 93 (30%) | 30 (37%) | 63 (27%) |  |
| ≥2 |  | 15 (4.8%) | 4 (4.9%) | 11 (4.7%) |  |
| CVD | 336 | 104 (31%) | 20 (28%) | 84 (32%) | 0.6 |
| Secondary Ca | 335 | 42 (13%) | 6 (8.5%) | 36 (14%) | 0.2 |
| Gleason Score 8-10 | 385 | 276 (72%) | 80 (83%) | 196 (68%) | 0.003 |
| Local therapy RP/RT | 419 | 123 (29%) | 24 (23%) | 99 (32%) | 0.091 |
| De Novo mHSPC | 413 | 313 (76%) | 93 (89%) | 220 (71%) | <0.001 |
| Visceral metastatis | 373 | 29 (7.8%) | 13 (15%) | 16 (5.6%) | 0.004 |
| High volume mHSPC | 341 | 192 (56%) | 65 (80%) | 127 (49%) | <0.001 |
| High risk mHSPC | 355 | 222 (63%) | 71 (87%) | 151 (55%) | <0.001 |
| Treatment mCRPC | 419 |  |  |  | <0.001 |
| ADT monotherapy |  | 1 (0.2%) | 0 (0%) | 1 (0.3%) |  |
| Chemotherapy |  | 56 (13%) | 12 (11%) | 44 (14%) |  |
| ARPI |  | 111 (26%) | 57 (54%) | 54 (17%) |  |
| Lu-PSMA |  | 12 (2.9%) | 2 (1.9%) | 10 (3.2%) |  |
| Radium |  | 5 (1.2%) | 3 (2.9%) | 2 (0.6%) |  |
| None/Other/NA |  | 234 (56%) | 31 (30%) | 203 (65%) |  |
| *^1^* Median (IQR); n (%) | | | | | |
| *^2^* Kruskal-Wallis rank sum test; Fisher’s exact test; Pearson’s Chi-square test | | | | | |
