# Supplementary material for: Androgen receptor pathway inhibitors vs. docetaxel chemotherapy for metastatic hormone-sensitive and first-line castration resistant prostate cancer
Source: World J Urol. 2024 Dec 28;43(1):51. doi: 10.1007/s00345-024-05388-1 (PMC11682002; doi:10.1007/s00345-024-05388-1)
Supplement: Supplementary file 4 — Supplementary Material 4: Table 2. Univariable und multivariable Cox regression models predicting time to metastatic castration resistant prostate cancer (mCRPC; A) and overall survival (OS; B) in metastatic hormone-sensitive prostate cancer (mHSPC) patients and progression-free survival (PFS, C) and OS (D) regarding sequential therapies in mCRPC: Abbreviation: HR: Hazard Ratio, CI: Confidence interval, androgen receptor pathway inhibitor (ARPI), ECOG: Eastern Cooperative Oncology Group. [file 345_2024_5388_MOESM4_ESM.docx]

|  | Univariable | | | Multivariable | | |
| --- | --- | --- | --- | --- | --- | --- |
| 1. Time to mCRPC | **HR** | **CI** | **p value** | **HR** | **CI** | **p value** |
| Docetaxel | **Ref.** | **-** | **-** | **Ref.** | **-** | **-** |
| All mHSPC: ARPI* | 0.49 | 0.37-0.66 | <0.001 | 0.96 | 0.58-1.58 | 0.9 |
| High volume mHSPC: ARPI* | 0.82 | 0.56-1.21 | 0.3 | 1.04 | 0.60-1.79 | 0.9 |
| 1. OS in mHSPC |  |  |  |  |  |  |
| Docetaxel | **Ref.** | **-** | **-** | **Ref.** | **-** | **-** |
| ARPI^+^ | 0.67 | 0.47-0.97 | 0.03 | 1.03 | 0.57-1.85 | 0.9 |
| High volume mHSPC: ARPI^+^ | 1.21 | 0.76-1.91 | 0.4 | 1.16 | 0.60-2.24 | 0.7 |
| 1. PFS in mCRPC |  |  |  |  |  |  |
| ARPI-ARPI | **Ref.** | **-** | **-** | **Ref.** | **-** | **-** |
| ARPI-Docetaxel* | 1.34 | 0.83-2.16 | 0.2 | 2.35 | 0.96-5.35 | 0.06 |
| Docetaxel-ARPI* | 0.93 | 0.61-1.42 | 0.7 | 0.77 | 0.39-1.52 | 0.4 |
| High volume mHSPC:  ARPI-Docetaxel* | 1.42 | 0.69-2.93 | 0.3 | 2.18 | 0.66-7.18 | 0.2 |
| High volume mHSPC: Docetaxel-ARPI* | 0.82 | 0.42-1.58 | 0.6 | 0.96 | 0.37-2.49 | 0.9 |
| 1. OS in mHSPC |  |  |  |  |  |  |
| ARPI-ARPI | **Ref.** | **-** | **-** | **Ref.** | **-** | **-** |
| ARPI-Docetaxel^+^ | 0.95 | 0.47-1.95 | 0.9 | 2.54 | 0.86-7.51 | 0.09 |
| Docetaxel-ARPI^+^ | 1.31 | 0.74-2.31 | 0.4 | 1.53 | 0.66-3.58 | 0.3 |
| High volume mHSPC:  ARPI-Docetaxel^+^ | 1.51 | 0.60-3.85 | 0.4 | 3.04 | 0.67-13.9 | 0.2 |
| High volume mHSPC: Docetaxel-ARPI^+^ | 1.04 | 0.45-2.37 | 0.9 | 1.29 | 0.34-4.88 | 0.7 |

Adjustment in multivariable Cox regression models was made for:

*Age at mHSPC, PSA at mHSPC, ECOG status, De Novo mHSPC, year of diagnosis, (+high volume disease)

^+^ Age at mHSPC, PSA at mHSPC, ECOG status, De Novo mHSPC, year of diagnosis, amount of systemic treatment lines, (+ high volume disease)
