# Supplementary material for: Androgen receptor pathway inhibitors vs. docetaxel chemotherapy for metastatic hormone-sensitive and first-line castration resistant prostate cancer
Source: World J Urol. 2024 Dec 28;43(1):51. doi: 10.1007/s00345-024-05388-1 (PMC11682002; doi:10.1007/s00345-024-05388-1)
Supplement: Supplementary file 4 [file 345_2024_5388_MOESM2_ESM.docx]

Supplemental Figure 2. Kaplan Meier curves depicting progression-free survival (PFS, A) and overall survival (OS) in metastatic hormone-sensitive prostate cancer (mHSPC, B) and OS in metastatic castration resistant prostate cancer patients (mCRPC, C) with initially high volume (HV) metastatic hormone-sensitive prostate cancer stratified according to sequential treatments with docetaxel (Doce) and androgen receptor pathway inhibitors (ARPI).
